# Supplementary material for: The Complete Mitochondrial Genomes of Two Rock Scallops (Bivalvia: Spondylidae) Indicate Extensive Gene Rearrangements and Adaptive Evolution Compared with Pectinidae
Source: Int J Mol Sci. 2023 Sep 8;24(18):13844. doi: 10.3390/ijms241813844 (PMC10531248; doi:10.3390/ijms241813844)
Supplement: Supplementary file 1 [file ijms-24-13844-s001.zip › ijms-2541129-supplementary.pdf]

Table S1. Gene annotations of the mtDNA of *S. versicolor*.

Gene	Strand	Location	Size (bp)	Start Codon	Stop codon	Intergenic nucleotides
Cox1	H	1-1560	1560	ATG	TAG	1
Nad4	H	1562-2845	1284	ATG	TAA	2
tRNA-*Leu1*	H	2848-2922	75			19
Cox2	H	2942-3612	671	ATA	TA	1945
Cox3	H	5558-6412	855	GTG	TAG	17
tRNA-*Thr*	H	6430-6500	71			1
Nad2	H	6502-7458	957	ATA	TAA	18
Nad4L	H	7477-7767	291	ATG	TAG	47
Cytb	H	7815-8936	1122	ATT	TAG	5
tRNA-*Trp*	H	8942-9009	68			1
Atp8	H	9011-9181	171	GTG	TAG	-1
tRNA-*Arg*	H	9181-9251	71			4
tRNA-*Pro*	H	9256-9322	67			13
tRNA-*Ile*	H	9336-9403	68			4
tRNA-*Ser1*	H	9408-9475	68			2
tRNA-*Leu2*	H	9478-9545	68			50
Nad3	H	9596-9898	303	ATG	TAA	32
tRNA-*Val*	H	9931-9996	66			37
tRNA-*Asp*	H	10034-10098	65			1870
tRNA-*Glu*	H	11969-12041	73			2311
tRNA-*Tyr*	H	14353-14425	73			644
tRNA-*Gly*	H	15070-15136	67			0
tRNA-*Gln*	H	15137-15207	71			401
rrnL	H	15609-17099	1491			34
tRNA-*Phe*	H	17134-17201	68			138
rrnS	H	17340-18274	935			0
tRNA-*Ala*	H	18275-18342	68			150
tRNA-*Met1*	H	18493-18561	69			615
tRNA-*Met2*	H	19177-19245	69			4706
Atp6	H	23952-24719	768	ATG	TAG	2
tRNA-*His*	H	24722-24789	68			11
tRNA-*Cys*	H	24801-24865	65			168
Nad6	H	25034-25555	522	ATT	TAA	5
tRNA-*Asn*	H	25561-25629	69			6
tRNA-*Lys*	H	25636-25701	66			4
tRNA-*Ser2*	H	25706-25774	69			91
Nad1	H	25866-26798	933	ATA	TAG	29
Nad5	H	26828-28525	1698	ATG	TAA	75

Table S2. Gene annotations of the mtDNA of *S. spinosus*.

Gene	Strand	Location	Size (bp)	Start Codon	Stop codon	Intergenic nucleotides
Cox1	H	1-1551	1551	ATG	TAA	9
Nad4	H	1561-2844	1284	TTG	TAG	3
tRNA-*Leu1*	H	2848-2921	74			19
Cox2	H	2941-3611	671	ATA	TA	1154
Cox3	H	4766-5620	855	GTG	TAA	2
tRNA-*Thr*	H	5623-5693	71			2
Nad2	H	5696-6652	957	ATA	TAA	18
Nad4L	H	6671-6961	291	ATT	TAG	56
Cytb	H	7018-8130	1113	ATT	TAG	1
tRNA-*Trp*	H	8132-8199	68			6
Atp8	H	8206-8379	174	ATG	TAA	225
tRNA-*Arg*	H	8605-8675	71			2
tRNA-*Pro*	H	8678-8743	66			6
tRNA-*Ile*	H	8750-8818	69			4
tRNA-*Ser1*	H	8823-8890	68			5
tRNA-*Leu2*	H	8896-8960	65			50
Nad3	H	9011-9313	303	ATA	TAA	11
tRNA-*Val*	H	9325-9390	66			8
tRNA-*Asp*	H	9399-9466	68			3847
tRNA-*Tyr*	H	13314-13384	71			1552
tRNA-*Gly*	H	14937-15002	66			6
tRNA-*Gln*	H	15009-15079	71			409
rrnL	H	15489-16948	1460			29
tRNA-*Phe*	H	16978-17045	68			134
rrnS	H	17180-18107	928			21
tRNA-*Ala*	H	18129-18195	67			125
tRNA-*Met*	H	18321-18388	68			4396
tRNA-*Glu*	H	22785-22859	75			84
Atp6	H	22944-23672	729	TTG	TAG	0
tRNA-*His*	H	23673-23739	67			14
tRNA-*Cys*	H	23754-23818	65			167
Nad6	H	23986-24510	525	ATG	TAG	10
tRNA-*Asn*	H	24521-24587	67			3
tRNA-*Lys*	H	24591-24657	67			6
tRNA-*Ser2*	H	24664-24731	68			87
Nad1	H	24819-25751	933	ATG	TAG	25
Nad5	H	25777-27474	1698	ATG	TAA	93

Table S3. List of AT content, AT skew, and GC skew of *S. versicolor* (*Sve*) and *S. spinosus* (*Ssp*).

Feature	(A+T)%		AT skew		GC skew	
	Sve	*Ssp*	*Sve*	*Ssp*	*Sve*	*Ssp*
Whole genome	57.9	58.3	-0.23	-0.21	0.39	0.34
PCGs	58.6	58.8	-0.38	-0.34	0.43	0.36
PCGs1	54.8	54.7	-0.20	-0.19	0.43	0.39
PCGs2	60.3	59.6	-0.42	-0.44	0.22	0.21
PCGs3	60.7	62.1	-0.49	-0.39	0.62	0.48
Atp6	57.8	59.9	-0.42	-0.44	0.42	0.44
Atp8	63.2	66.7	-0.48	-0.52	0.46	0.45
Cox1	57.9	58.5	-0.31	-0.32	0.27	0.24
Cox2	58.8	58.4	-0.25	-0.20	0.35	0.24
Cox3	56.3	59.7	-0.38	-0.32	0.42	0.33
Cytb	60.8	61.3	-0.31	-0.24	0.38	0.29
Nad1	59.7	56.9	-0.43	-0.39	0.45	0.38
Nad2	58.9	60.2	-0.40	-0.37	0.50	0.44
Nad3	56.4	59.1	-0.44	-0.34	0.44	0.42
Nad4	58.4	58.0	-0.43	-0.40	0.51	0.40
Nad4L	58.4	56.0	-0.39	-0.46	0.52	0.50
Nad5	58.7	59.7	-0.40	-0.34	0.47	0.42
Nad6	57.1	50.5	-0.36	-0.36	0.48	0.41
tRNAs	46.5	48.7	-0.17	-0.15	0.23	0.24
rrnL	54.9	54.5	-0.02	-0.01	0.25	0.26
rrnS	53.7	54.0	0.02	0.07	0.22	0.20

Table S4. Codon and relative synonymous codon usage (RSCU) of 13 protein-coding genes (PCGs) in the mtDNA of *S. versicolor* and *S. spinosus*.

Amino Acid	Codon	Count (RSCU)		Amino Acid	Codon	Count (RSCU)	
		Sve	*Ssp*			*Sve*	*Ssp*
Phe	UUU	291.0(1.74)	273.0(1.67)	Ala	GCU	117.0(2.39)	94.0(2.07)
	UUC	44.0(0.26)	54.0(0.33)		GCC	20.0(0.41)	24.0(0.53)
Leu	UUA	158.0(1.94)	184.0(2.22)		GCA	18.0(0.37)	44.0(0.97)
	UUG	187.0(2.30)	148.0(1.79)		GCG	41.0(0.84)	20.0(0.44)
	CUU	90.0(1.11)	85.0(1.03)	Gly	GGU	112.0(1.13)	116.0(1.21)
	CUC	9.0(0.11)	13.0(0.16)		GGC	18.0(0.18)	24.0(0.25)
	CUA	23.0(0.28)	34.0(0.41)		GGA	59.0(0.60)	54.0(0.56)
	CUG	21.0(0.26)	33.0(0.40)		GGG	206.0(2.09)	190.0(1.98)
Ile	AUU	140.0(1.74)	153.0(1.70)	Arg	CGU	30.0(1.38)	32.0(1.51)
	AUC	21.0(0.26)	27.0(0.30)		CGC	5.0(0.23)	7.0(0.33)
Met	AUA	73.0(0.79)	88.0(1.09)		CGA	20.0(0.92)	13.0(0.61)
	AUG	111.0(1.21)	73.0(0.91)		CGG	32.0(1.47)	33.0(1.55)
Val	GUU	231.0(2.21)	223.0(2.14)	Tyr	UAU	129.0(1.62)	99.0(1.33)
	GUC	23.0(0.22)	31.0(0.30)		UAC	30.0(0.38)	50.0(0.67)
	GUA	63.0(0.60)	65.0(0.63)	His	CAU	57.0(1.58)	53.0(1.51)
	GUG	102.0(0.97)	97.0(0.93)		CAC	15.0(0.42)	17.0(0.49)
Ser	UCU	96.0(2.31)	92.0(2.01)	Gln	CAA	12.0(0.57)	14.0(0.58)
	UCC	14.0(0.34)	19.0(0.42)		CAG	30.0(1.43)	34.0(1.42)
	UCA	17.0(0.41)	32.0(0.70)	Asn	AAU	60.0(1.74)	51.0(1.57)
	UCG	15.0(0.36)	10.0(0.22)		AAC	9.0(0.26)	14.0(0.43)
	AGU	39.0(0.94)	54.0(1.18)	Lys	AAA	17.0(0.34)	27.0(0.58)
	AGC	10.0(0.24)	18.0(0.39)		AAG	84.0(1.66)	66.0(1.42)
	AGA	30.0(0.72)	42.0(0.92)	Asp	GAU	68.0(1.84)	54.0(1.59)
	AGG	111.0(2.67)	99.0(2.16)		GAC	6.0(0.16)	14.0(0.41)
Pro	CCU	74.0(2.23)	71.0(2.03)	Glu	GAA	22.0(0.37)	27.0(0.47)
	CCC	21.0(0.63)	23.0(0.66)		GAG	96.0(1.63)	87.0(1.53)
	CCA	18.0(0.54)	21.0(0.60)	Cys	UGU	77.0(1.56)	79.0(1.60)
	CCG	20.0(0.60)	25.0(0.71)		UGC	22.0(0.44)	20.0(0.40)
Thr	ACU	63.0(2.42)	65.0(2.39)	Trp	UGA	26.0(0.40)	31.0(0.48)
	ACC	9.0(0.35)	9.0(0.33)		UGG	105.0(1.60)	98.0(1.52)
	ACA	17.0(0.65)	15.0(0.55)	*	UAA	5.0(0.83)	6.0(1.00)
	ACG	15.0(0.58)	20.0(0.73)		UAG	7.0(1.17)	6.0(1.00)

Table S5. Best fit partitions and substitution models.

	Set Partition (Conducted by PartitionFinder 2)	Best Model for BI (Selected by PartitionFinder 2)	Best Model for ML (Selected by ModelFinder)
Best Partition CDS genes (BIC= 267077.43)	*Atp6* 1th	TRN+G	TIM3+F+G4
	Atp6 2th	TVM+G	TIM2+F+I+G4
	Atp6 3th	TIM+G	TVM+F+G4
	Cox1-2-3 1th	GTR+G	TIM2+F+G4
	Cox1-2-3 2th	TVM+G	HKY+F+G4
	Cox1-2-3 3th	TIM+I+G	TVM+F+G4
	Cytb 1th	TRN+G	TVM+F+G4
	Cytb 2th	TVM+G	TN+F+I+G4
	Cytb 3th	TRN+G	GTR+F+G4
	Nad1-2-3-4-4L-5-6 1th	GTR+I+G	TIM+F+G4
	Nad1-2-3-4-4L-5-6 2th	TVM+G	TVM+F+G4
	Nad1-2-3-4-4L-5-6 3th	TIM+I+G	TIM2+F+I+G4
Best Partition CDS genes (BIC= 17474.65)	*rrnS-rrnL*	GTR+G	TIM+F+G4

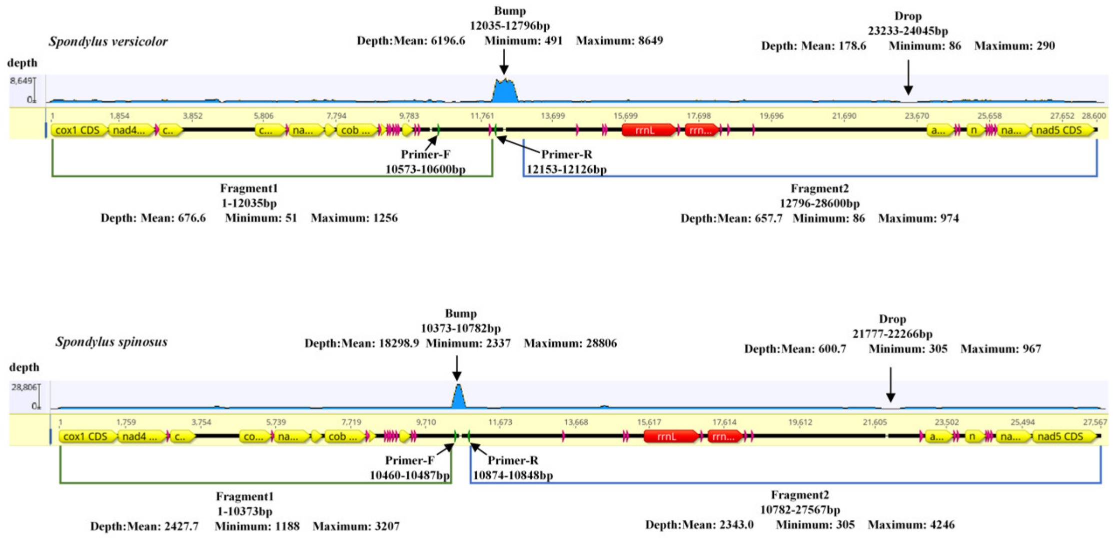

Figure S1. The coverage plots of two mitogenomes.

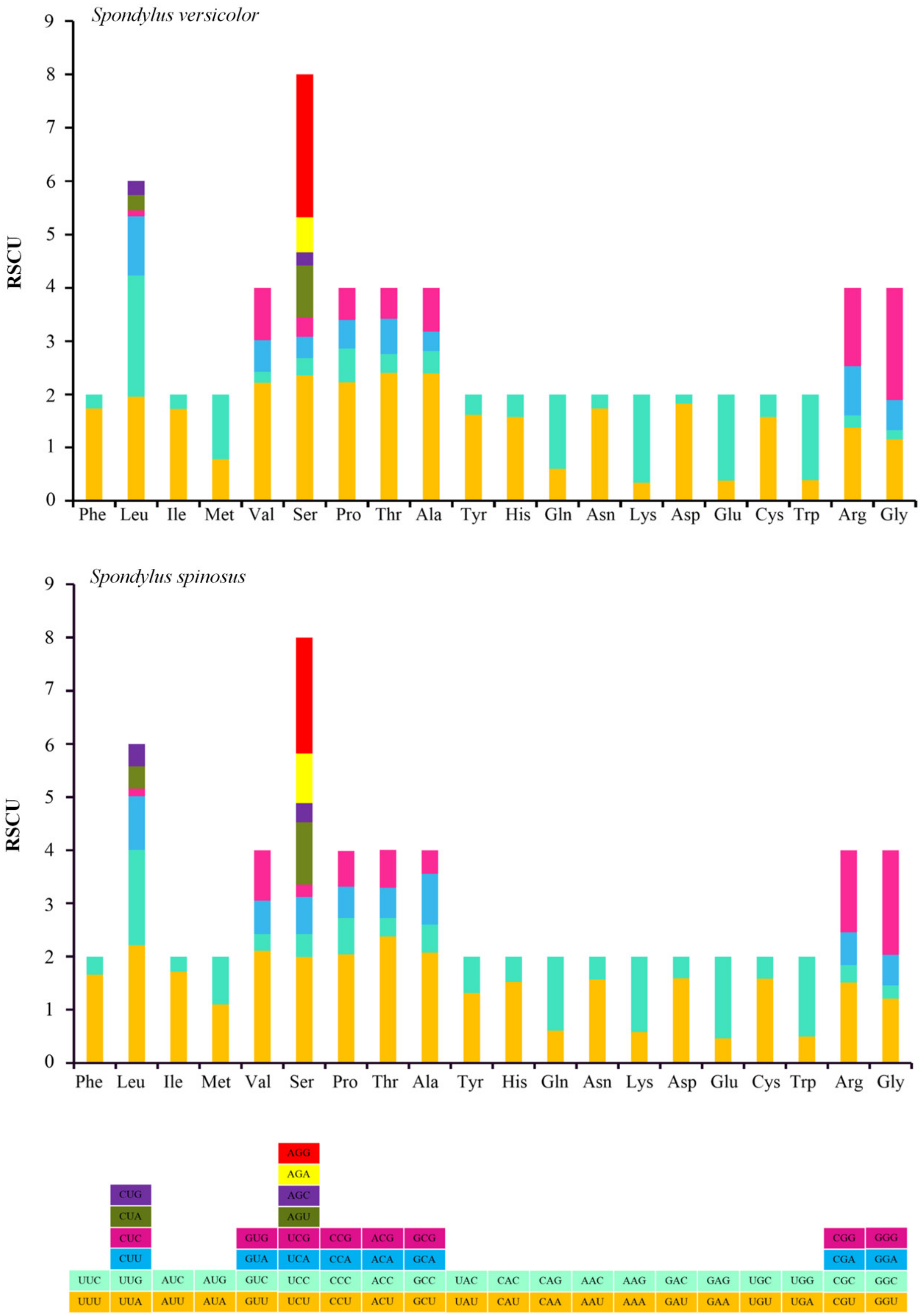

Figure S2. Relative synonymous codon usage (RSCU) of mitochondrial genomes for *S. versicolor* and *S. spinosus*.

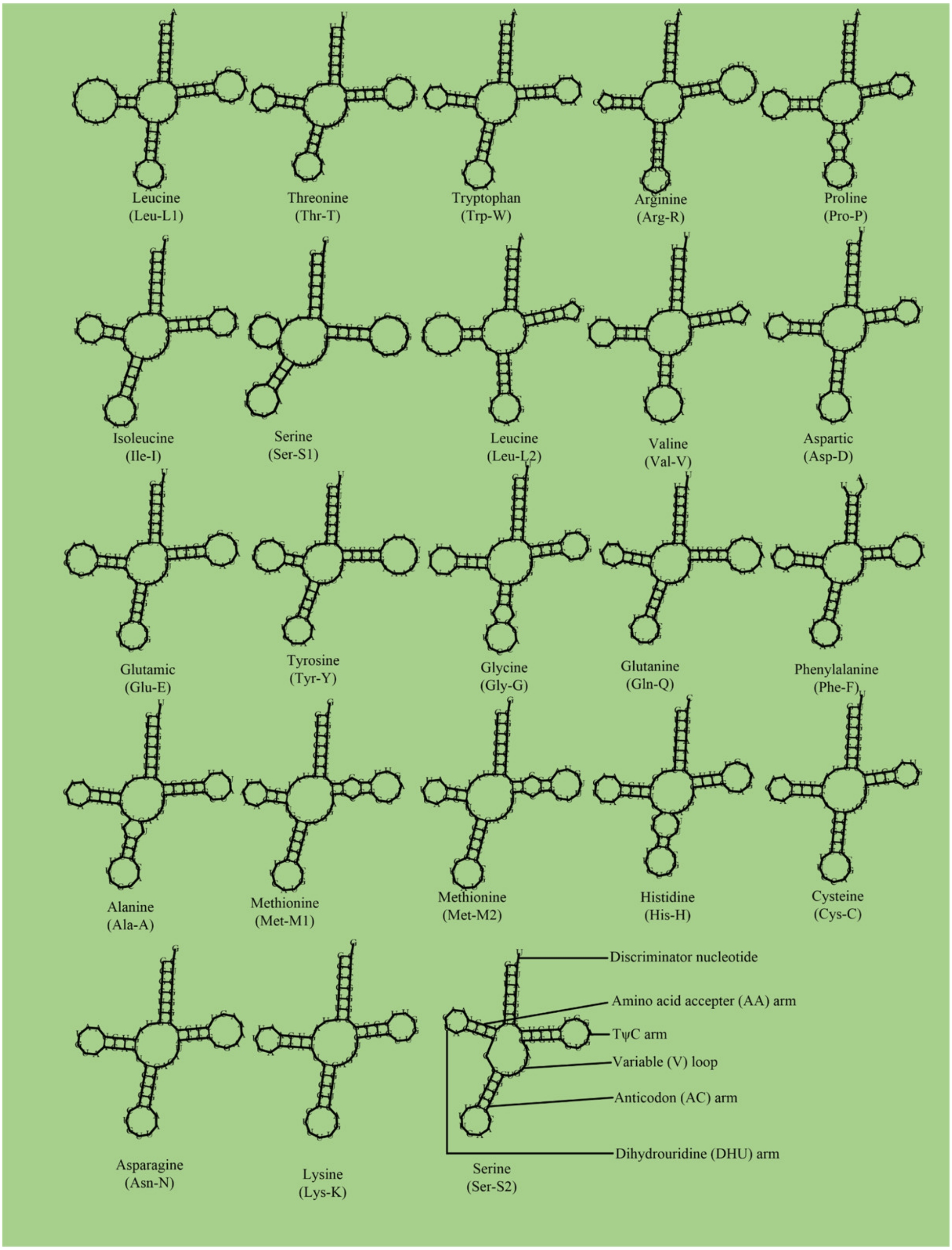

Figure S3. Inferred secondary structures of 23 transfer RNAs represented by *S. versicolor*.
